# Cattle transporters' attitudes, indigenous knowledge, and current practices towards animal welfare, occupational well-being, and operational challenges: A survey of five regions in Ghana

**DOI:** 10.1016/j.heliyon.2024.e27317

**Published:** 2024-03-05

**Authors:** J.W.S. Mogre, F. Adzitey, G.A. Teye, P.T. Birteeb

**Affiliations:** a Council for Scientific and Industrial Research, Animal Research Institute, P. O. Box AH 20, Achimota-Accra, Ghana; b University for Development Studies, Faculty of Agriculture, Food and Consumer Sciences, Department of Animal Science, P.O. Box TL 1882, Tamale, Ghana

**Keywords:** Animal welfare attitudes, Occupational risk, Transporters, Stockmanship, Ghana, Transporters' attitudes and knowledge, Indigenous knowledge and cattle transportation

## Abstract

Ghana is a significant cattle producer in Africa with an estimated cattle population of 3 million in 2020 [1]. The role of transporters in linking farms to markets and slaughterhouses is crucial in the livestock value chain. However, cattle transportation subjects the animals to high levels of stress, compromising their welfare, and transporters face challenges such as long working hours and harsh conditions.

The objective of this study was to explore current practices, indigenous knowledge, and operational risks pertaining to animal welfare among transporters.

The study was conducted in five regions connected by the N16, N10, and N6 Highway, the primary route for cattle transportation from the Ghana-Burkina Faso border to the coastal capital of Accra. A total of 78 transporters participated in the study.

Out of the 78 participants in this study, the majority of transporters were young adults (56.5%) with secondary school education (45.9%) and 0–5 years of experience (37.2%). The average distance and time for transporting cattle was 528 km and 18 h, respectively. The transporters highlighted significant challenges encountered during cattle transport, including feed and water shortage, particularly prominent during the dry season (32.1%), as well as the occurrence of diseases and mortality, particularly high during the rainy season (41%). Furthermore, vehicular breakdowns were a common issue reported by transporters, with a substantial majority (88.5%) experiencing breakdowns during their last 10 trips. The results showed that most transporters (84.7%) had limited knowledge of animal welfare and had not received any formal education on livestock transportation. Instead, they gained experience as assistants on other trucks before becoming drivers. The findings of this study highlight the need for improved welfare standards for cattle during transportation and formal training programs for transporters in animal welfare and livestock transportation.

## 1 Introduction

Transporters play a crucial role in the livestock industry, as they are responsible for the movement of animals from farms to marketing centers and meat processing facilities, thereby establishing a vital link within the livestock value chain. The transportation of live animals is known to be stressful and can have a direct impact on animal welfare and food safety and quality [1]. Globally, cattle are transported by various means of transport and over different terrains, including herding, trucking, rail, and over water bodies. Challenges in ensuring cattle welfare have persisted over time and continue to be relevant today [2].

Livestock transporters must perform several roles aside just transporting, which include the basics of stockmanship and animal husbandry [3]. Factors that impact animal welfare during transportation include the microclimate, loading density, duration of transport, quality of transport, animal behavior and the animals fitness for transport [2,4]. To enhance animal welfare during transportation, all parties involved must be informed about the animal and how to preserve their welfare [5,6]. Planning journeys, choosing suitable vehicles, and allowing adequate space for animal movement are critical [2].

The role of transporters in animal welfare and One-Health is important to consider, as the One-Health approach recognizes the interconnectedness of animal, human, and environmental health [7]. Professional transporters are under pressure to drive for long periods and irregular schedules, exposing them to extended shifts, sleep restrictions, postural fatigue, noise-vibration, sedentary lifestyle, unhealthy diet, diesel exhaust fumes, and other occupational stressors, increasing the risk of road accidents [8]. Adopting One-Health principles can lead to improved health and welfare of farm animals, reduced occupational hazards, and a more sustainable understanding of human-animal interactions.

This study aimed to investigate the current practices and indigenous knowledge regarding animal welfare and operational risks faced by cattle transporters in Ghana. The hypotheses of this study were: 1) the operational risks faced by cattle transporters in Ghana during transportation negatively impacts the welfare of the cattle and the safety of the transporters; 2) the current practices and indigenous knowledge of cattle transporters in Ghana with respect to animal welfare are insufficient to ensure optimal conditions for the cattle during transportation; and 3) Cattle transportation in Ghana is inadequate and negatively impacts the health and welfare of the animals.

## 2 Materials and methods

The study was carried out from February 2019 to March 2020 and used a cross-sectional survey and an observational study to collect data from a sample of cattle transporters in Ghana.

**Fig. 1:**
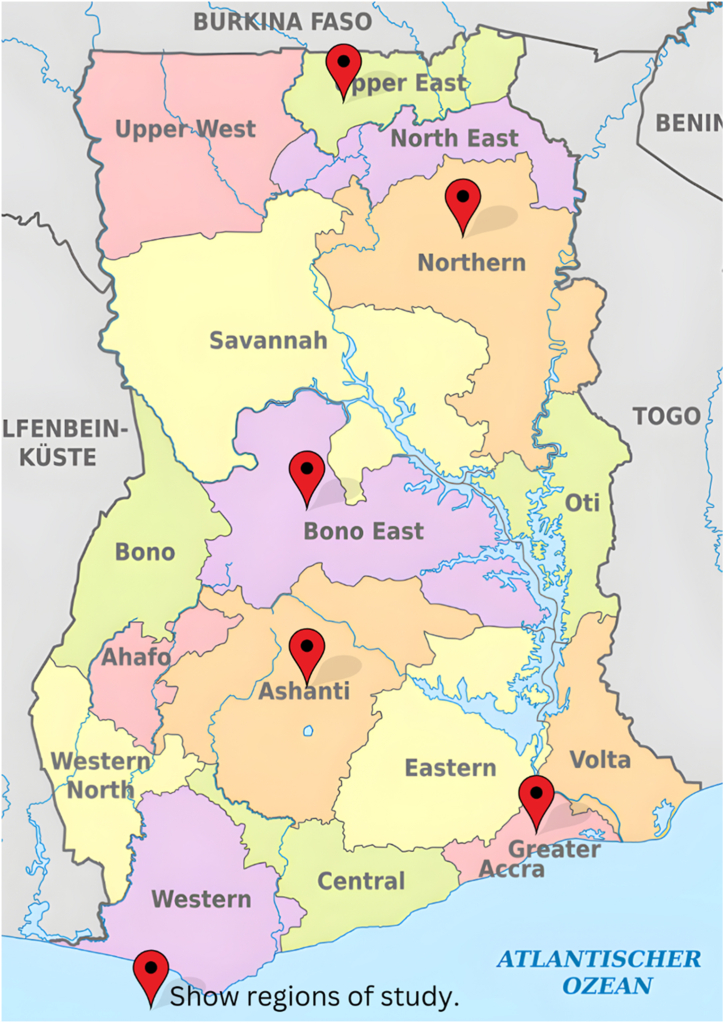
Map of Ghana showing the regions where the study was undertaken. Wikipedia contributors, 'Regions of Ghana,' Wikipedia, The Free Encyclopedia, last modified January 24, 2024, https://en.m.wikipedia.org/wiki/Regions_of_Ghana (accessed January 24, 2024).

The study was conducted in 5 administrative regions in Ghana as shown in Fig. 1. Ghana is situated on the west coast of Africa with a total area of 238,540 Km2 [9]. Ghana is found approximately between Latitude: 7° 57′ 9.97″ N Longitude: −1° 01′ 50.56″ W [10]. The country is divided into 16 administrative regions and has a population of 30.8 million (GSS, 2021).

### 2.1 Study design

#### 2.1.1 Survey

A cross-sectional survey was conducted using a combination of closed- and open-ended questions to gather both quantitative and qualitative data. The survey was conducted with 78 cattle transporters in Ghana. The participants were selected using convenience sampling. At cattle loading and offloading sites, we engaged with leaders of the cattle transporters. After explaining the purpose and data collection process, these leaders designated representatives to introduce our research team to willing transporters for interviews. The information was collected via an on-site interview lasting between 40 and 50 min, and efforts were made to minimize bias by keeping the main objectives of the study undisclosed to the participants, as per [11]. The sample size was determined based on the project time and the willingness of transporters to provide information. The transporters in the study plied the N16, N10, and N6 Highway, which is the primary route for cattle transportation in Ghana. The route starts from the Ghana-Burkina Faso border at Paga in the north and ends at the coastal capital of Accra in the south.

Purposive sampling was used to select five administrative regions for the study. A total of seventy-eight (78) transporters were sampled, thus, 5 in Upper East Region, 20 in Northern Region, 8 in Bono East, 25 in Ashanti Region, and 20 in Greater Accra Region. Transporters were interviewed at the main cattle markets and slaughterhouses in each region.

The variation in the number of respondents among the five administrative regions in our study of cattle transporters was likely influenced by several factors, including population size and density, economic activity, and sampling bias. Specifically, regions with larger populations or higher levels of economic activity may have had more respondents.

The Design and Development of the Questionnaire and Measurement Scales for the Survey.

In order to effectively collect data for this survey, a questionnaire was carefully crafted with a dual focus: ensuring the precision and relevance of the questions, and minimizing any undue stress on the participants, who are primarily cattle transporters. This approach was essential to gather accurate data while being considerate of the unique challenges faced by the respondents in their line of work.A preliminary study was carried out in January 2019, during which a draft questionnaire was applied to ten transporters. These participants were excluded from the final sample. The results of the pilot study were utilized to refine and finalize the questionnaire, which consisted of 64 questions divided into eight sections. The first section, entitled "Demographic Characteristics," aimed to gather information on the participants' demographic details. The subsequent sections, including "Freedom from Hunger, Malnutrition, and Thirst," "Freedom from Fear and Distress," "Freedom from Physical and Thermal Discomfort," "Freedom from Pain, Injury, and Disease," and "Freedom to Express Normal Patterns of Behaviour," sought to assess the transporters' observations of the animals they transport and the animals' responses to the stressful environments they encounter. The seventh section, "Transporters' Indigenous Knowledge of Animal Welfare, Attitudes, and Practices, and Occupational Risk and Hazards," examined the participants' views on animal welfare and their experiences with occupational risks and hazards.

Participant observations were conducted to assess loading and unloading procedures, vehicle suitability, and sanitary conditions within cattle transportation. A total of 78 cattle transporters were observed while loading or unloading cattle at the sites. Our study covered both dry and wet seasons to account for potential seasonal variations in challenges faced by transporters. We employed a detailed observational checklist to document specific aspects, including areas such as lighting, flooring, animal welfare, vehicle conditions, and driver competence. Unloading areas were also inspected for animal behavior and signs of distress. The data collection process involved systematic observations, ensuring a comprehensive evaluation of cattle transportation practices from a welfare and safety standpoint.

### 2.2 Data collection

Data were collected using a combination of methods, including surveys and observations. The survey included closed-ended questions on the main challenges faced by transporters, the comfort level of animals during transport, and the effectiveness of animal handling equipment used by transporters. The survey also included open-ended questions for more detailed information on the challenges faced by transporters and suggestions for improvements in the animal transportation process in Ghana.

### 2.3 Data analysis

Data were analyzed using descriptive statistics, and inferential statistics to identify patterns and trends in the data. This helped to draw conclusions about the business of transporting animals in Ghana, the main challenges faced by transporters, and the comfort level of animals during transport. All data were analyzed using Statistical Package for Social Sciences (SPSS version, 2013).

### 2.4 Ethical considerations

The study was conducted in accordance with the ethical principles of the Declaration of Helsinki. All participants were informed about the study and provided written consent to participate. The study was also approved by the institutional review board. All data collected were kept confidential, and the identities of the participants were kept anonymous.

## 3 Results

### 3.1 General assessment

The study conducted in five regions of Ghana allowed researchers to gather general information about cattle transport characteristics and demographic data of the transporters.

**Fig. 2:**
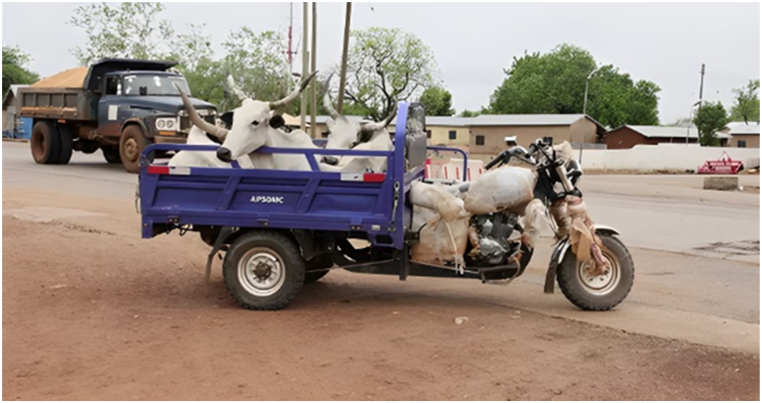
Motor tricycle used for cattle transportation, with cattle secured with ropes in constrained postures to restrict mobility.

**Fig. 3:**
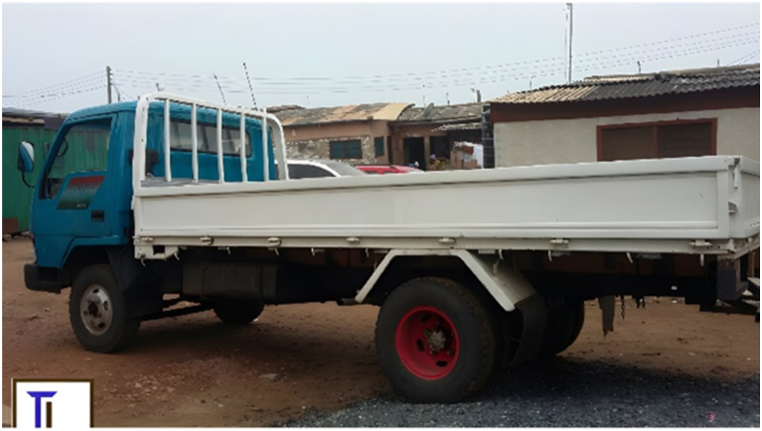
Example of LCV's used in transporting cattle.

**Fig. 4:**
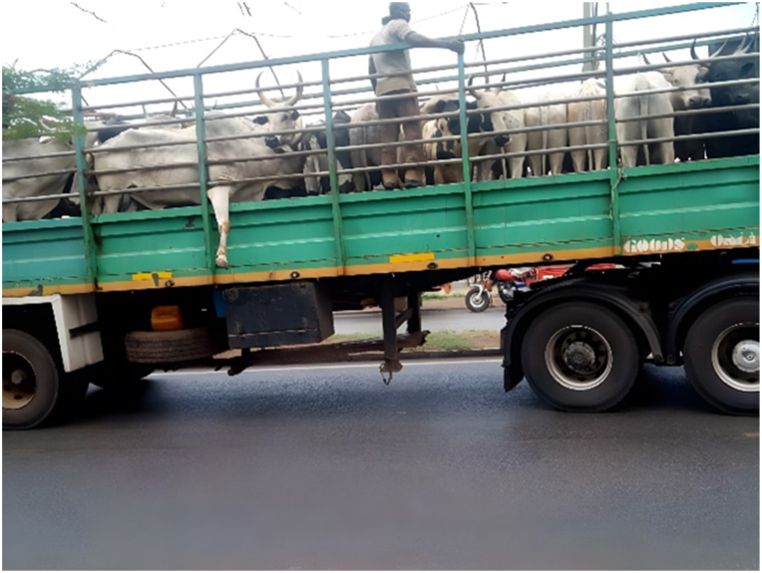
Overcrowding of cattle in truck carriage during transportation in Ghana.

**Fig. 5:**
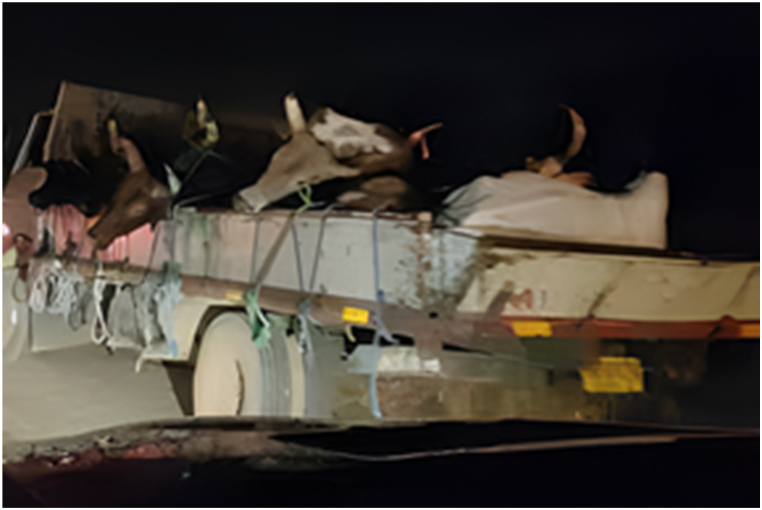
An LCV transporting cattle at night along the Accra motorway , with cattle secured with ropes in constrained postures. photo credit GhanaWeb.com.

The main destinations for the transporters were the Ashanti and Greater Accra regions. The majority of transporters used Semi Trailer Trucks (56.4%), followed by Light Commercial Vehicles (LCVs) (12.8%) and motor tricycles (19.2%) shown in Fig. 2, Fig. 3, Fig. 4 respectively. Only 39.5% of the vehicles inspected were considered fit for cattle transportation. In this study, "fit for transport vehicles" were operationally defined as vehicles characterized by sufficient space, appropriate ventilation, non-slip flooring, and strategically placed floor openings to facilitate drainage. The average number of cattle carried in a vehicle was 27, with a maximum of 150 and a minimum of 2.Fig. 5

**Fig. 6:**
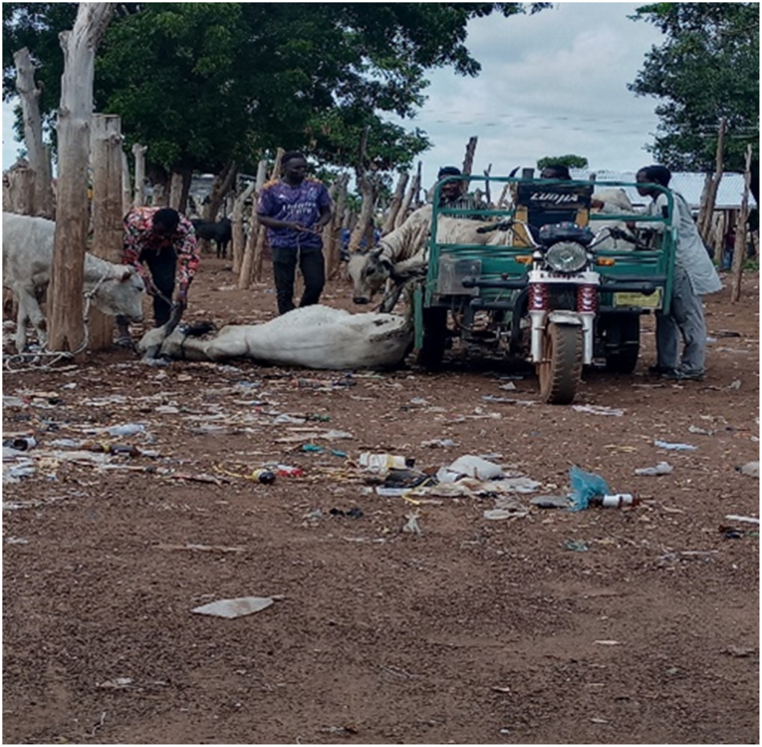
Post-transportation immobilization and compromised ambulation observed in bovine subjects following vehicular transportation, resulting in dragging during unloading process.

**Fig. 7:**
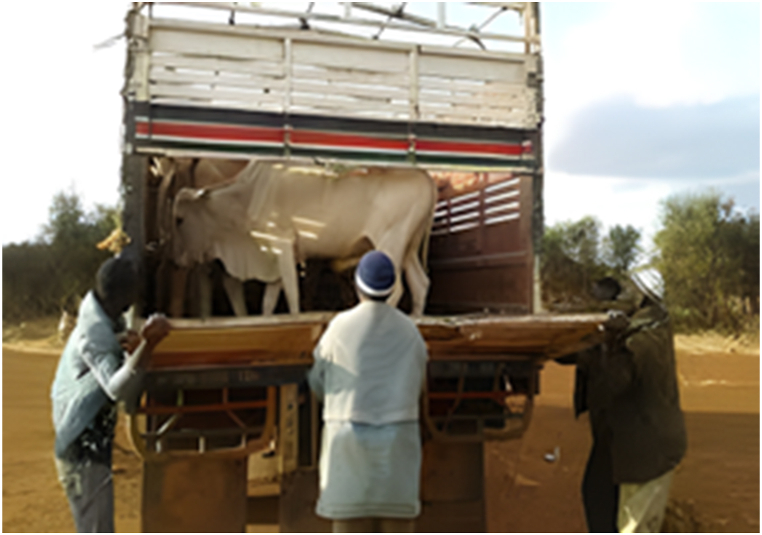
Cattle being confined within the vehicle for an extended journey.

**Fig. 8:**
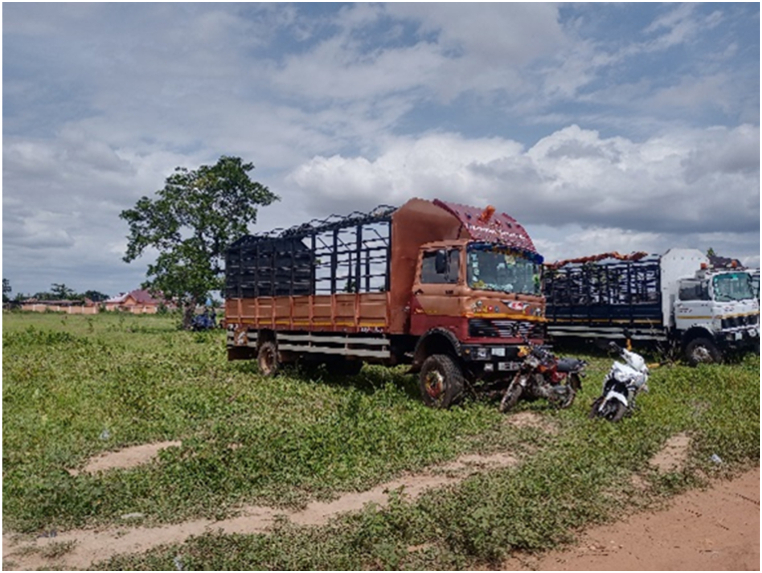
Queue of vehicles awaiting their turn at the Tamale cattle market, preparing to load cattle for the southward journey.

Table 1 shows that all transporters were male, with the majority being over the age of 30 years and having an age range of 41–45 years. The educational backgrounds of the transporters varied, with 29.5% having no formal education, 44.9% having a secondary school education and 10.3% having tertiary education. The average years of experience as a transporter was 10.7 years with a maximum of 27 years. Results from this study showed that the business of transporting animals in Ghana often revolves around butchers and farmers who congregate their animals and find a transporter willing to take the animals to a desired destination. Therefore, one vehicle could have several animals belonging to different customers on a trip. The minimum distance travelled with cattle was 150 Km and the maximum distance was 720 Km (the average distance was 528 KM). On average, transporters spent 18 h in transit. The minimum hours spent was 12 h and the maximum hours spent on a trip was 30 h. Each vehicle involved in cattle transportation had a single driver who drove for the entirety of the trip. The number of rest stops drivers took varied based on the distance they would cover, ranging from one to three rest stops. It was observed that during the process of loading and unloading animals onto vehicles, the predominant method employed was physically securing and lifting the animals into the vehicles (51.3%), whereas the utilization of a loading ramp was observed in 48.7% of instances. Fig. 6, Fig. 7, Fig. 8 show various instances of cattle being loaded and offloaded without a ramp. All cattle transported in this study were beef cattle, encompassing multiple breeds including N'Dama, West African Short Horn, Gudali, White Fulani, and various crossbreeds among them.

**Table 1:** Demographic indicators of 78 transporters.

Demographic	
Indicator	Transporters
Age (%)	
Below 18	0
18–40	56.5
41–60	43.5
61-Above	0
	100
Education (%)	
None	29.7
Primary	14.9
Secondary	45.9
Tertiary	9.5
	100
Years of Experience (%)	
0–5	37.2
6–10	19.2
11–15	12.8
15- Above	30.8
	100

Table 2 presents a comprehensive overview of the observational assessment, providing valuable insights into the presence or absence of specific factors pertaining to vehicle suitability and animal handling during cattle transport in Ghana. The data offer a detailed snapshot of the assessment, shedding light on key aspects of interest in the context of enhancing the welfare and transportation conditions of cattle.

**Table 2:** Observational assessment of vehicle and animal handling during cattle Transport in Ghana.

Assessment of vehicles and animal handling	Yes		No	
	Number of Vehicles	%	Number of Vehicles	%
Vehicle fit for purpose of transporting cattle	31	39.70	47	60.30
Anti-slip-on vehicle floor	47	60.30	31	39.70
Adequate air vents on vehicle.	75	96.20	3	3.80
Drainage holes on floor	49	62.80	29	37.20
Carrier partitioned	13	16.70	65	83.30
Loading ramp	47	60.30	31	39.70
Transporters use handling equipment	55	70.50	23	29.50

Majority (52%) of transporters reported that they paid levies to regulatory bodies such as customs, police, revenue authority, district/municipal/metropolitan assemblies, and veterinary officers. These levies are collected as tax and are supposed to ensure that livestock and other animals being transported are healthy.

**Fig. 9:**
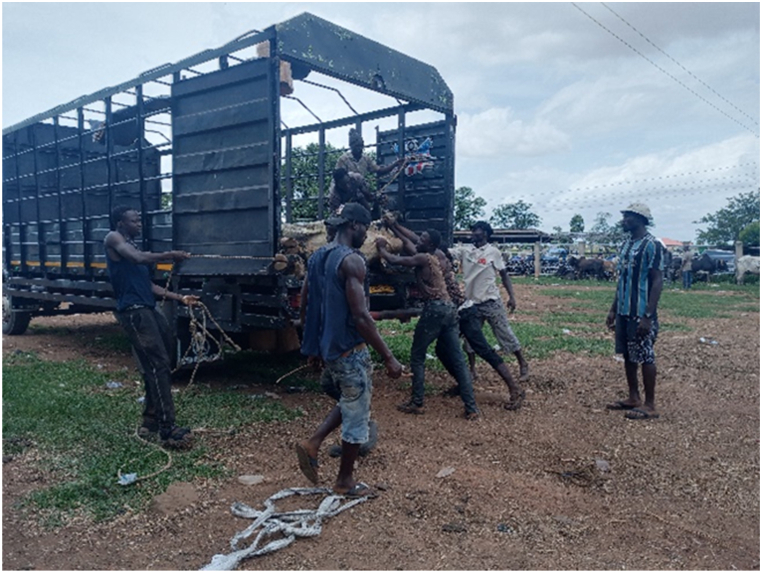
A collaborative effort by a team of seven individuals to manually lift and load a bull weighing over 300 kg into a truck for transportation purposes.

**Fig. 10:**
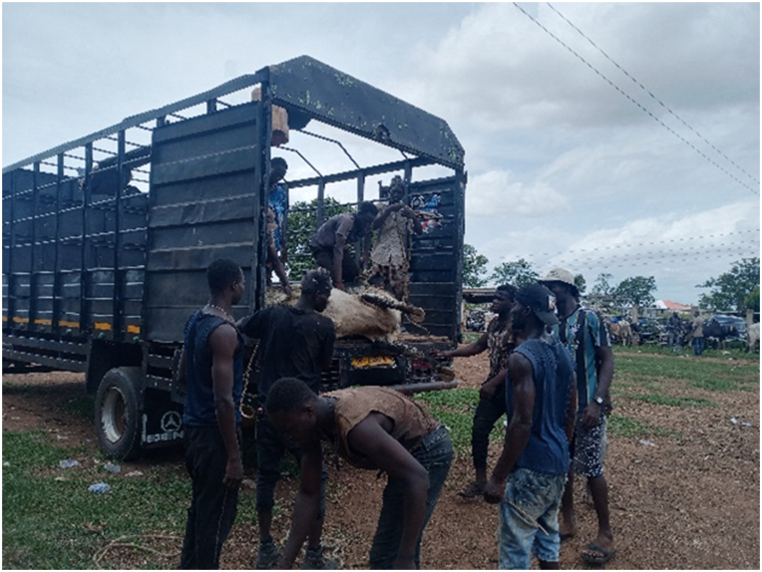
Men securing a bull in a truck to prevent escape due to inadequate cattle transportation infrastructure.

**Fig. 11:**
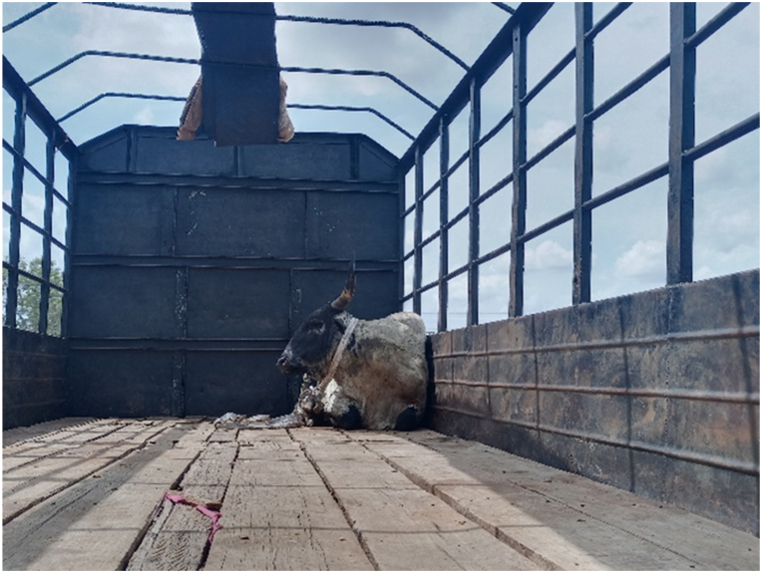
First bull securely tied up in a truck, awaiting the loading of additional cattle. The truck has a capacity to transport 16-20 cattle.

Fig. 9, Fig. 10, Fig. 11 shows cattle being loaded onto a truck for an 18 h journey. The main problems transporters faced, in order of magnitude and effect on their activities were.

• Lack of access to water and feed for animals in transit (42.3%): A predominant concern among transporters was the lack of access to water and animal feed during the course of transportation. This issue, accounting for 42.3% of the responses, is indicative of a considerable challenge with tangible repercussions on the welfare of the transported animals.
• Financial losses (28.2%): Transporters incur financial losses due to various factors such as delays, vehicle breakdowns, or unexpected expenses. These losses impact profitability and sustainability of the transportation business.
• Police extortion (24.4%): Transporters face challenges related to corruption and extortion by law enforcement officials. Such practices disrupt smooth transportation operations and add unnecessary costs.
• Poor sanitation of loading and offloading areas (2.6%): there was an absence of measures in place for the proper disposal of animal waste.
• Cost of repair and maintenance (1.3%): referring to incurred costs related to vehicle repair and maintenance
• Lack of fit for purpose vehicles (1.2%): limited access to fit-for-purpose vehicles for the transport of cattle.

Transporters reported seasonal variations in the problems they encountered. In the dry season (November–April) the paramount problem was scarcity of feed and water for the animals (32.1%). Disease and mortality (41%) were the paramount problem encountered in the rainy/wet season.

Among the 78 transporters who were questioned about their vehicle maintenance practices, the activities included oil changes, fluid checks and replacements, tire rotations, filter replacements, brake inspections, and other routine upkeep activities. It was found that 21% of the transporters serviced their vehicles on a quarterly basis, 19% serviced their vehicles following each long journey, 36% serviced their vehicles twice annually, and 24% serviced their vehicles once annually. The types of livestock species transported by the transporters were also examined. It was found that 72% of transporters only carried cattle, while the remaining 28% transported a combination of cattle, sheep, and goats. In terms of the sizes of the cattle being transported, 79.5% of transporters mixed cattle of varying sizes, while the remaining 20.5% transported cattle of similar size.

As seen in Table 2, the study examined the fitness of vehicles for transporting cattle, as well as the ventilation and comfort of the animals during transport. The results revealed that the proportions of fit and unfit vehicles were as follows: 39.70% fit for the purpose of transporting cattle and 60.30% not fit. In terms of specific vehicle features, 60.30% had anti-slip-on vehicle floors, and 96.20% had adequate ventilation. Additionally, 62.80% had drainage holes on the floor, while only 16.70% were partitioned. Notably, 60.30% of the vehicles had loading ramps. After loading, 21.80% of the animals were comfortable, while 52.60% of the animals appeared visibly sick or diseased. Furthermore, 70.50% of the transporters reported using ropes and whips as handling equipment.

The study revealed that a majority of transporters (84.7%) lacked knowledge of the concept of animal welfare. Only 15.3% of the participants demonstrated an understanding of animal welfare, with 91% of this subgroup defining it as the provision of food and medical attention to animals. Additionally, 77.3% of transporters reported no training in animal welfare, and the remaining 22.7% who had received training reported being taught basic animal health checks, healthy animal selection, and proper animal restraint techniques.

The majority of the transporters (77.3%) were not trained in animal welfare, with training being imparted by older transporters after several years of experience as a driver's mate. The remaining 22.7% who had received training reported being taught basic animal health checks, healthy animal selection, and proper animal restraint techniques.

**Fig. 12:**
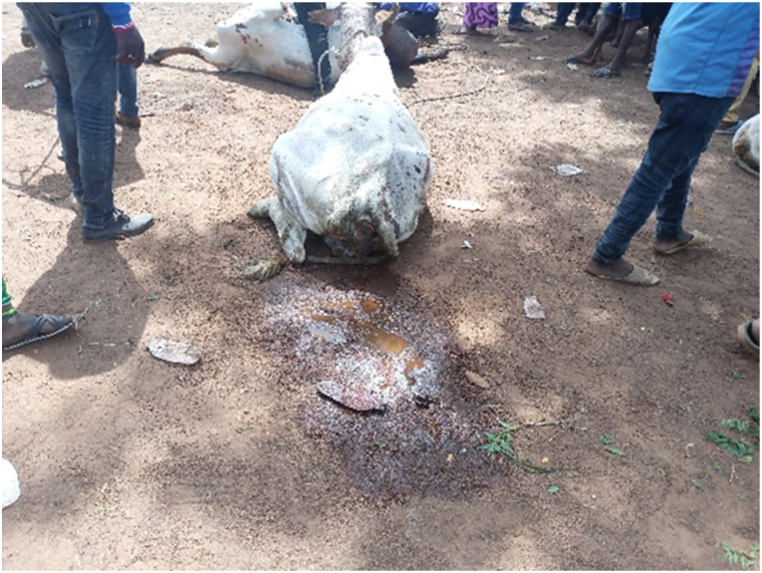
Manifestation of fatigue, stress, and fear-induced urination in debilitated cattle.

**Fig. 13:**
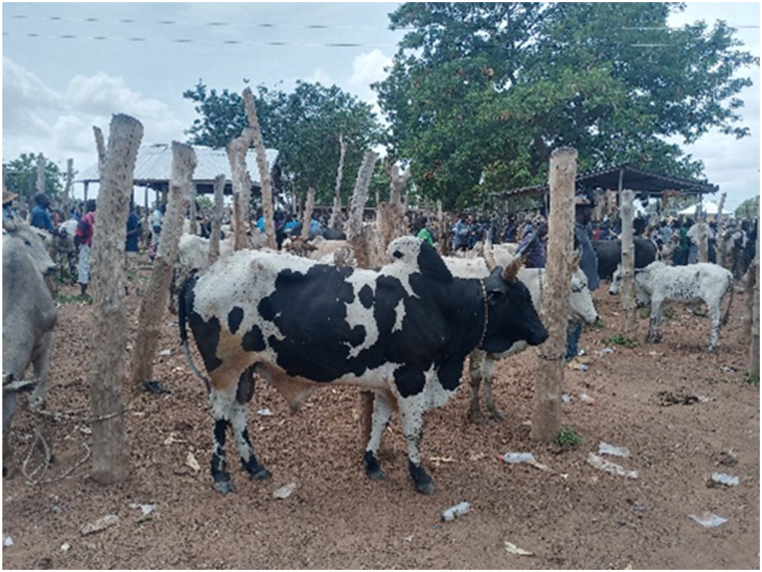
Cattle tethered to stakes in preparation for an extended 18-hour transport journey.

**Fig. 14:**
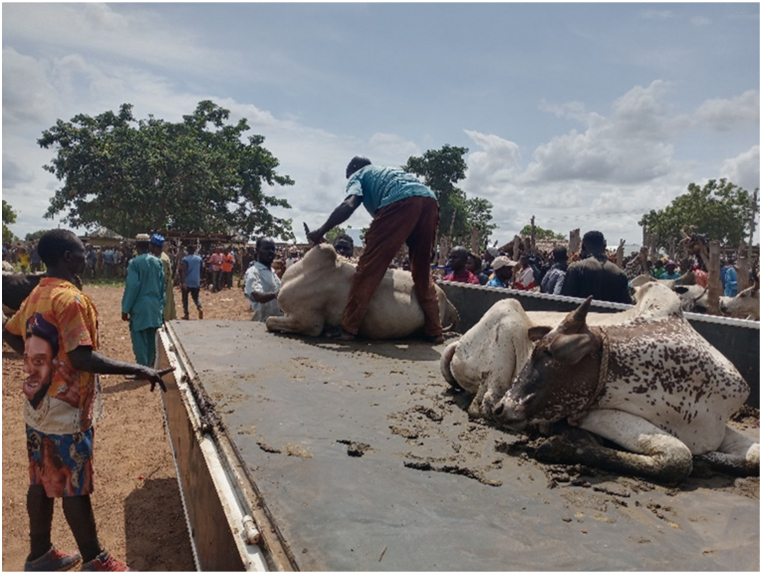
Cattle being offloaded from a truck. Cattle are tied in a constrained and contorted posture during the extended journey.

**Fig. 15:**
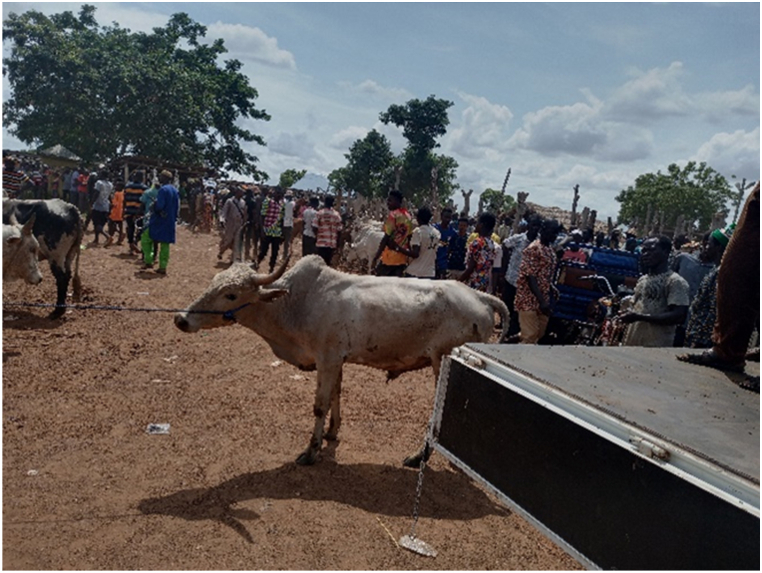
Cattle being assisted into a standing position after falling from a vehicle, requiring manual dragging.

The results showed that the majority (65.4%) of transporters provided feed to their cattle during transport. The most commonly used feed was cut grass (89%), with no consideration given to the type or age of the grass. A small proportion (11%) of transporters provided rice or corn residue as feed. In terms of hydration, 61.5% of transporters provided water to their animals during transport. Of these, 58.3% carried water for the animals and 41.7% stopped to fetch water from natural water bodies along the route. Feeding was typically carried out once per trip. In the course of this study, it was noted that upon reaching their destination, the animals under observation exhibited symptoms indicative of dehydration and hunger. This was further evidenced by the behavior of some animals, which included nibbling at wooden posts. The signs of dehydration observed included lethargy, tightening of the skin, drying of mucous membranes and eyes, and sunken eyes. This can be observed in Fig. 12, Fig. 13, Fig. 14, Fig. 15.

**Fig. 16:**
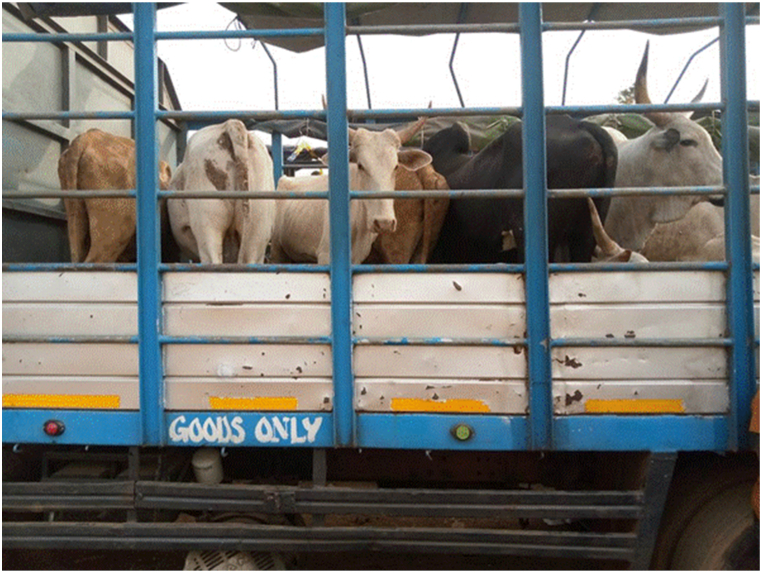
Cattle successfully loaded and secured in the truck for transportation.

According to the transporters in this study, animals typically showed signs of fear and distress during transport (see Fig. 16). To judge the comfort of the animals, the transporters relied on two primary factors: calmness (89.6%) and visual assessment of available space (10.4.

The results showed that 39.7% of the vehicles inspected were considered fit for this purpose, while the remaining 60.3% were not fit. On average, each vehicle had three additional attendants in addition to the driver. The maximum number of attendants recorded was eight, while the minimum was one. The number of attendants accompanying livestock during transportation varied according to the type of vehicle utilized. On average, Semi-Trailer Trucks were accompanied by three attendants, while Light Commercial Vehicles (LCVs) had two attendants, and motor tricycles had one attendant. It is noteworthy that in certain instances, Semi-Trailer Trucks carried cattle from multiple farmers and intermediaries, resulting in an increased number of attendants. This arrangement aimed to assign a dedicated individual within the carriage area to closely monitor the specific animals under their care, thereby mitigating the risks of potential fatalities or injuries. Additionally, the study found that a significant proportion (88.5%) of transporters reported incidents of vehicle breakdown while transporting cattle. However, most transporters did not have contingency protocols in place for such situations. When a vehicle broke down, the transporters typically arranged for a mechanic to fix the vehicle while the cattle were either confined within the carriage area or secured with ties, as depicted in Fig. 4, Fig. 5, Fig. 6, Fig. 7, Fig. 8, Fig. 9, Fig. 10, Fig. 11. The majority of vehicles (81%) that broke down were repaired within 24 h, and only 26.3% of vehicles provided bedding for the animals. These findings suggest that the transportation of cattle may be associated with a high incidence of vehicle breakdowns and that there may be a lack of contingency plans in place to address such situations.

Almost all transporters (94.7%) reported that they were able to identify sick animals before loading them onto their vehicles eventhough they had no formal training in disease identification.

The results showed that for every ten journeys, 67% of transporters reported losing between 0 and 5 animals, while 17.5% lost 6–10 animals with the average mortality rate among transporter who could estimate mortality being 15.4%. However, a significant proportion (16%) of responders were unable to provide an approximate death toll. Additionally, 74% of transporters reported that animals died during transport.

According to the transporters, a substantial majority (80.3%) reported transporting visibly pregnant animals. When confronted with situations where animals fell ill or sustained injuries during transit, the transporters' responses varied. Notably, 79.2% of the transporters indicated that they did not take any measures to assist the affected animals. A minority (6.5%) reported loosening restraints to enhance the comfort of the animals. Additionally, some transporters described efforts to isolate the animal as much as possible (9.1%), while a few (1.3%) increased the speed of their vehicle to expedite arrival at the destination. In cases where an animal was perceived as likely to die, 9.1% of the transporters chose to euthanize the animal. Furthermore, a significant number of transporters (74.4%) mentioned carrying animals with broken legs.

Injury to animals during transport was reported by 89.7% of transporters, with the most common cause being aggression or fighting (75%). Other causes of injury included trying to escape from the moving vehicle (2.5%), loading and off-loading (5%), trampling or being weak or sick due to overcrowding (15%), and bad roads (2.5%).

According to the transporters in this study, the main means of handling aggressive animals during transport were beating (71.8%) and the use of restraints (ropes, 28.2%). The transporters also reported that different breeds of cattle had varying levels of difficulty in terms of handling. The easiest breed to handle was the White Fulani, while the most difficult to handle was the Ndama.

According to the transporters' accounts, a notable 39% admitted to taking no action when animals exhibited such signs. Conversely, 37% reported stopping the transport to allow the animals time to rest. A smaller group, constituting 21%, took measures to separate the weaker animals from the rest. In more critical situations, 3% of the transporters resorted to pouring water on animals in need of resuscitation. Furthermore, the study revealed that aggression and mating behaviors were prevalent during transport. A significant 82.1% of transporters observed fighting among animals. Additionally, 70.5% of the transporters reported witnessing male animals attempting to mate with female animals while in transit.

## 4 Discussion

The primary aim of this study was to explore the practices, knowledge, and challenges faced by cattle transporters in Ghana, with a particular focus on animal welfare and transporter safety. Our findings reveal a significant gap in the understanding and application of animal welfare principles among transporters, with a majority displaying limited knowledge and inadequate practices.

The cattle in this study were transported for an average of 18 h which is longer than the EU's preference of as short as possible, but they did not exceed the 24-h limit set by North America [12,13].

Proper loading ramps were not present at most cattle markets, resulting in the use of physical force to lift the animals into the vehicles. This most probably caused significant stress for both the animals and handlers, as it required significant effort and multiple workers to complete the task. Other authors also observed a similar issue [14]. The loading and offloading process has been found to cause significant stress in animals [1], and the absence of proper ramps in this study exacerbated this stress. Additionally, transporters reported that fees levied by regulators were excessive and burdensome. Similar research work done in the Ashanti region of Ghana also noted that cattle transporters experienced delays and high fees at security checkpoints and from veterinary or quarantine officials [14]. In a similar vein, a study documented comparable issues with fees levied by police at various checkpoints in Nigeria [15]. These conditions may have contributed to the reported mortality rates recorded in this study which were significantly higher than those reported by others for cattle [16,17].

Most transporters did not segregate animals by size or sex during transport, leading to numerous injuries and deaths. The negative significant impact on animal welfare during transport arises from a combination of factors, including in-transit injuries resulting from mating and other aggressive behaviors, the transportation of sick or injured animals, the detrimental effects of overcrowding, inadequate provision of feed and water, as well as the refusal of transporters to adhere to recommended rest stops [18]. Additionally, none of the transporters adhered to recommended stocking densities, which has been shown to have detrimental effects on livestock and negatively impact their welfare [[19], [20], [21]]. In instances where small ruminants were transported alongside cattle, a makeshift upper deck was created to accommodate the small ruminants. Transporters often carried both small and large ruminants on the same vehicles in an effort to maximize profits per trip. The upper compartment effectively separated the sheep and goats from the cattle, eliminating the risk of trampling.

Despite being able to identify sick animals before loading them onto their vehicles, transporters admitted to transporting sick animals. Many transporters were observed loading visibly sick or diseased animals during this study. Transporters frequently had animals dying in transit and generally did nothing for animals that became sick or injured during transport.

The findings of this study shed light on the attitudes and practices of Ghanaian cattle transporters in relation to animal welfare. The majority (84.7%) of the transporters had no knowledge of the concept of animal welfare, which is a concerning issue given the important role they play in the transport of live animals. A mere 15.3% of the transporters were able to provide a definition of animal welfare, which primarily consisted of providing feed and medical care to the animals The informal training system used by the transporters is also a matter of concern. The majority (77.3%) received no training in animal welfare from older transporters, which leaves them lacking in crucial knowledge and skills for properly caring for the animals during transport. The remaining 22.7% who received some form of animal welfare training only received basic training in topics such as purchasing healthy animals, conducting basic health checks, and proper animal restraint techniques.

It is important to note that the provision of adequate training in animal welfare is crucial for the welfare of the animals during transport [22,23]. The lack of knowledge and training in this area could lead to poor handling practices, causing undue stress and injury to the animals. Moreover, inadequate animal welfare practices can also negatively impact the reputation of the industry and result in decreased consumer confidence in the products [[24], [25], [26]].

### 4.1 Ghanaian cattle characteristics

The absence of a consistent national policy on cattle breeds for beef or milk production in Ghana has resulted in a diverse array of breeds and crossbreeds being transported [27]. The lack of widely accepted breeding objectives for cattle farmers has led to a focus on breeding for size and growth rate, with little attention given to breeding for docility [28]. This situation has s implications for the welfare of the animals during transport, since docile animals are easier to handle and experience less stress during transportation [29]. In this study transporters found the White Fulani breed of cattle to be the easiest to handle. In a study by Ref. [30], the White Fulani breed was found to be moderately docile. However, others found that the Friesian/White Fulani cross was more susceptible to injuries and stress during transportation compared to the Brahman/Gudali cross, based on the effects of different road conditions on rectal temperature, behavior, and traumatic injuries during transportation of different crosses of temperate or tropical breeds of heifers [31].

### 4.2 Animal welfare and seasonal considerations

According to the transporters', the main challenge they faced was ensuring that there was enough water and feed available for the animals during transit. Withholding feed and water has been shown to lead to weight loss through both water and tissue loss, depending on the duration of the fast [1], resulting in economic losses for animal owners at slaughter , the transporters' ranking of this challenge as the highest indicates a potential gap in their appreciation of the broader context of animal welfare and the various influential factors within their control that can significantly impact the welfare of the cattle during transportation. While it is understandable that the availability of water and feed during transit is indeed a crucial aspect of ensuring animal welfare, the transporters did not consider other important factors that also play significant roles. One notable aspect that transporters overlooked is the occurrence of mating during transit, which can lead to injuries and stress among the animals. The transportation of sick or injured animals, without proper care and veterinary attention, exacerbates their conditions (increases the risk of mortality). Furthermore, the high stocking density, or overcrowding, within the transport vehicles is another significant concern, as it most likely contributes to heightened stress levels, fighting between animals and greater vulnerability to injuries and diseases leading to mortality [32].

The high incidence of sick/diseased animals in the rainy season has been confirmed by several studies that mention the prevalence of parasites and diseases is higher in the rainy season compared to the dry season [33]; [34,35]. Thus, animals are more likely to be sick during the rainy season. The added stress of transportation increases the likelihood that sick animals will die during or shortly after transport. Seasonal availability of feed for cattle is a challenge faced by the entire livestock industry [36,37], and transporters may prioritize delivery of the animals over the provision of feed. The main objective of the transporter seems to deliver the animal alive, rather than in optimal condition.

Injuries to animals during transport were primarily due to poor vehicle structures, lack of partitioning, and poor stockmanship by transporters. The main method of handling aggressive animals was through beating animals with sticks. Welfare challenges associated with rough handling have been well documented ^14^^.^In Ghana it is extensively documented that cattle farmers frequently opt to sell weakened and ailing cattle in order to minimize potential financial losses [[38], [39], [40]]. Consequently, it is reasonable to posit that the mortality rates reported by participants of this study may be attributed to a combination of the pre-existing health conditions of the animals, which are aggravated by the challenging handling and transportation conditions encountered by these debilitated or unwell animals during transit.

### 4.3 Vehicle conditions and welfare

Many of the vehicles included in this study were deemed unsuitable for cattle transportation due to their lack of sufficient space for animal movement, inadequate ventilation, absence of non-slip floors, and inadequate openings for effective drainage. Moreover, these vehicles exhibited a high frequency of breakdowns during the transportation of animals. When problems during transit were encountered, transporters would wait for up to 24 h for repairs to be completed, causing significant stress for the animals confined in the carriage. This issue aligns with the findings of previous studies [41,42], which highlighted the lack of specialized vehicles for animal transport in most countries in Africa. The high frequency of vehicular breakdown in this study raises serious concerns about animal welfare, as continuous vehicular movement is necessary to ensure proper ventilation, and time spent for transit is a factor in mortality-related losses [43].

Properly designed and equipped vehicles play a crucial role in ensuring the welfare of animals during transportation. The concept of fit-for-transport vehicles encompasses various elements that contribute to the welfare of the transported animals. These elements include sufficient space for animal movement, appropriate ventilation systems, non-slip flooring to prevent injuries, and effective drainage mechanisms to maintain cleanliness and minimize health risks.

Recent studies have emphasized the importance of utilizing specialized vehicles specifically designed for animal transport. Researchers such [44,45] have investigated the impact of vehicle design on animal welfare outcomes. These studies have highlighted the benefits of vehicles equipped with adjustable partitions, appropriate flooring materials, and efficient ventilation systems, resulting in reduced stress, injuries, and mortality rates during transportation.

Additionally, research conducted that focused on the role of vehicle suspension systems in minimizing the negative effects of transport vehicles on animal welfare [46]. These studies have emphasized the importance of well-maintained suspension systems in reducing stress, discomfort, and potential injuries to the transported animals.

By utilizing fit-for-transport vehicles that adhere to recommended design standards and maintenance practices, transporters can significantly enhance animal welfare outcomes during transit. Implementing guidelines and regulations that promote the use of appropriate vehicles and encouraging ongoing research and innovation in vehicle design are vital steps toward ensuring optimal welfare during transportation.

### 4.4 Interconnectedness of human, animal, and environmental health

Considering the interconnectedness of human, animal, and environmental health, commonly referred to as One Health, it is vital to recognize the broader impact of welfare concerns during animal transport [47]. The transportation of cattle, in particular, has the potential to affect the health and welfare of both the transported animals and the humans involved in the process [48].

Moreover, the transport process itself can serve as a means for the spread of pathogens, thereby increasing the risk of disease transmission within and between animal populations [49].

Proper management of animal transport is of utmost importance to ensure the welfare of animals and to minimize risks to both human and animal health. The findings of the study indicate a significant risk not only to the welfare of the animals but also to the welfare of humans and the environment.

One observed practice during cattle transport that warrants attention is the carriage of multiple stockmen in the trucks alongside the cattle. While this practice is rooted in the desire to ensure the safety and welfare of the animals during transit, it raises several concerns. Firstly, the practice of having multiple stockmen within the confined space of the transport vehicle may not only pose challenges for the comfort and welfare of the animals but also create occupational health and safety risks for the stockmen themselves. Prolonged exposure to the confined environment, noise, and animal-related stress factors can contribute to occupational stress and health issues [50]. The practice of carrying multiple stockmen does not align with modern best practices in animal transport. This practice indicates the dire need for comprehensive training of stockmen and transporters [51].

#### 4.4.1 Considerations and future Directions

This study offers important insights into cattle transportation practices in Ghana, serving as a foundational exploration in this area. While the findings are significant, we acknowledge certain considerations for future research.

1. Scope of Transporters' Responses: The insights gained from the transporters, derived through surveys and interviews, provide a valuable perspective on current practices. While these responses are essential for understanding real-world scenarios, future studies might benefit from a more diverse array of data collection methods to further enrich our understanding.
2. Observational Study Approach: The observational aspect of this study provided a practical overview of transportation conditions. This approach was chosen for its real-world applicability and to establish a baseline understanding. Future studies could build on this by implementing more detailed observational protocols, thereby enhancing the depth of data collected.
3. Foundation for Detailed Research: This study serves as an initial step in exploring cattle transportation in Ghana. It opens several avenues for more focused research, particularly in areas identified as critical. Future studies could delve deeper into specific aspects, employing more targeted methodologies.

This study, as an initial foray into the field, sets the stage for more comprehensive future research. It aims not only to inform but also to inspire further exploration into the multifaceted aspects of cattle transportation, animal welfare, and transporter safety.

## 5 Conclusion

In conclusion, this study underscores the urgent need for improvements in cattle transportation practices in Ghana. Inadequate infrastructure, including the lack of loading ramps, overcrowding, the transport of sick and injured animals, and poor animal handling skills, are probably associated with higher mortality rates. Transporters' limited knowledge of animal welfare highlights the importance of formal training to enhance animal welfare, prompting transporters to prioritize animal welfare beyond mere survival. Vehicle conditions, characterized by frequent breakdowns and inadequate features, must be addressed to reduce stress, injuries, and mortality during transportation. Recognizing the interconnectedness of human, animal, and environmental health (One Health) underscores the importance of comprehensive management, including addressing practices like carrying multiple stockmen in transport vehicles Future efforts should focus on infrastructure enhancement, training, and vehicle improvements, all while considering the broader implications for One Health.

## Declaration of funding

This research did not receive any specific funding.

## Data availability Statement

The data that support this study will be shared upon reasonable request to the corresponding author.

## CRediT authorship contribution statement

**J.W.S. Mogre:** Writing – review & editing, Writing – original draft, Project administration, Methodology, Investigation, Formal analysis, Data curation, Conceptualization. **F. Adzitey:** Writing – original draft, Validation, Methodology, Conceptualization. **G.A. Teye:** Supervision, Project administration, Methodology, Conceptualization. **P.T. Birteeb:** Writing – review & editing, Validation, Software, Methodology, Data curation.

## Declaration of competing interest

The authors declare that they have no known competing financial interests or personal relationships that could have appeared to influence the work reported in this paper.

## References

[1] Schwartzkopf-Genswein K., Ahola J., Edwards-Callaway L., Hale D., Paterson J. (2016). Symposium PAPER: transportation issues affecting cattle well-being and considerations for the future. Prof. Anim. Sci..

[2] Nielsen S.S., Alvarez J., Bicout D.J. (2022). Welfare of cattle during transport. EFSA J..

[3] Rushen J., Passillé AM de (2017). Improving Animal Welfare: A Practical Approach.

[4] Schuetze S.J., Schwandt E.F., Maghirang R.G., Thomson D.U. (2017). REVIEW: transportation of commercial finished cattle and animal welfare considerations. Prof. Anim. Sci..

[5] Nocella G., Hubbard L., Scarpa R. (2010). Farm animal welfare, consumer willingness to pay, and trust: results of a cross-national survey. Appl Econ Perspect Policy.

[6] Hernandez E., Fawcett A., Brouwer E., Rau J., Turner P.V. (2018). Speaking up: veterinary ethical responsibilities and animalwelfare issues in everyday practice. Animals.

[7] Cox J. (2022). https://www.wellbeingintlstudiesrepository.org/hw_onehealth/5/.

[8] Valadez-Noriega M., Estévez-Moreno L.X., Rayas-Amor A.A., Rubio-Lozano M.S., Galindo F., Miranda-de la Lama G.C. (2018). Livestock hauliers' attitudes, knowledge and current practices towards animal welfare, occupational wellbeing and transport risk factors: a Mexican survey. Prev. Vet. Med..

[9] Agble R., Bader E., Solal-Céligny A., Palma G., Dop M.C. (2009). https://www.moh.gov.gh/wp-content/uploads/2016/02/Nutrition-Country-Profile-Ghana.pdf.

[10] Kumi-Boateng B., Ziggah Y.Y. (2020). A 3D Procrustean approach to Transform WGS84 Coordinates to Ghana war Office 1926 reference Datum. Ghana Min J.

[11] Daros R.R., Hötzel M.J., Bran J.A., LeBlanc S.J., von Keyserlingk M.A.G. (2017). Prevalence and risk factors for transition period diseases in grazing dairy cows in Brazil. Prev. Vet. Med..

[12] EFSA (2016). https://www.bmel.de/SharedDocs/Downloads/EN/_Animals/position-paper-eu-legislation-animal-transport.pdf?__blob=publicationFile&v=2.

[13] Schwartzkopf-Genswein K.S., Faucitano L., Dadgar S., Shand P., González L.A., Crowe T.G. (2012). Road transport of cattle, swine and poultry in North America and its impact on animal welfare, carcass and meat quality: a review. Meat Sci..

[14] Frimpong S., Gebresenbet G., Bosona T., Bobobee E., Aklaku E., Hamdu I. (2012). Animal supply and Logistics activities of abattoir chain in Developing countries : the case of Kumasi abattoir , Ghana. J. Serv. Sci. Manag..

[15] Filani M.O. (2005). http://ir.library.ui.edu.ng/handle/123456789/998.

[16] Simova V., Voslarova E., Vecerek V., Passantino A., Bedanova I. (2017). Effects of travel distance and season of the year on transport-related mortality in cattle. Anim. Sci. J..

[17] Malena M., Voslářová E., Kozák A. (2007). Comparison of mortality rates in different Categories of pigs and cattle during transport for slaughter. Acta Vet Brno.

[18] Warren L.A., Mandell I.B., Bateman K.G. (2010). An audit of transport conditions and arrival status of slaughter cattle shipped by road at an Ontario processor. Can. J. Anim. Sci..

[19] Dalmau A., Temple D., Llonch P. (2009). Application of the welfare Quality® protocol at pig slaughterhouses Article in animal welfare. Anim. Welf..

[20] Broom D. (2001).

[21] Whiting T.L. (2000). Comparison of minimum space allowance standards for transportation of cattle by road from 8 authorities. Can. Vet. J..

[22] Wilhelmsson S., Andersson M., Hemsworth P.H., Yngvesson J., Hultgren J. (2023). Human-animal interactions during on-farm truck loading of finishing pigs for slaughter transport. Livest. Sci..

[23] Descovich K., Li X., Sinclair M., Wang Y., Phillips C.J.C. (2019). The effect of animal welfare training on the knowledge and attitudes of abattoir stakeholders in China. Animals.

[24] de Boer J., Aiking H. (2022). Considering how farm animal welfare concerns may contribute to more sustainable diets. Appetite.

[25] Alonso M.E., González-Montaña J.R., Lomillos J.M. (2020). Consumers' concerns and perceptions of farm animal welfare. Animals.

[26] Vizzier Thaxton Y., Christensen K.D., Mench J.A. (2016). Symposium: animal welfare challenges for today and tomorrow. Poult Sci.

[27] Okantah S. (2009). A review of studies on breed evaluation and genetic improvement of cattle in Ghana. Ghana J. Agric. Sci..

[28] Abdulai I.A., Dongzagla A., Ahmed A. (2023).

[29] Bates K.E., Weaber R.L., Bormann J.M. (2014). Temperament can be an indicator of feedlot performance and carcass merit in beef cattle. Kansas Agric Exp Stn Res Reports.

[30] Adedibu I.I., M S. (2017). Evaluation of Temperament and Morphometric Traits in white Fulani and Simmental X Sokoto Gudali cattle. Nigerian J. Anim. Sci..

[31] Minka N.S., Ayo J.O. (2018). Effects of different road conditions on rectal temperature, behaviour and traumatic injuries during transportation of different crosses of temperate/tropical breeds of heifers. Anim. Prod. Sci..

[32] Van Engen N.K., Coetzee J.F. (2018). Effects of transportation on cattle health and production: a review. Anim Heal Res Rev.

[33] Marufu M.C., Chimonyo M., Mapiye C., Dzama K. (2011). Tick loads in cattle raised on sweet and sour rangelands in the low-input farming areas of South Africa. Trop. Anim. Health Prod..

[34] Catley A., Osman J., Mawien C., Jones B.A., Leyland T.J. (2002). Participatory analysis of seasonal incidences of diseases of cattle, disease vectors and rainfall in southern Sudan. Prev. Vet. Med..

[35] Waruiru R.M., Kyvsgaard N.C., Thamsborg S.M. (2000). The prevalence and Intensity of Helminth and Coccidial Infections in dairy cattle in Central Kenya. Vet. Res. Commun..

[36] Akapali M., Ansah T., Abdul-Rahman, Alenyorege B., Baatuuwie B.N. (2018). Seasonal changes in pasture biomass and grazing behaviour of cattle in the Guinea Savanna agroecological zone of Ghana. African J Range Forage Sci.

[37] Konlan S.P., Ayantunde A.A., Dei H.K., Avornyo F.K. (2014). Evaluation of existing and potential feed resources for ruminant production in northern Ghana. Int Livest Res Inst Rep.

[38] Mockshell J., Ilukor J., Birner R. (2014). Providing animal health services to the poor in Northern Ghana: Rethinking the role of community animal health workers?. Trop. Anim. Health Prod..

[39] Adams F., Yankyera K.O. (2014). Socio-economic characteristics of Subsistent small ruminant farmers in three regions of northern Ghana. Asian J Appl Sci Eng.

[40] Nuvey F.S., Kreppel K., Nortey P.A. (2020). Poor mental health of livestock farmers in Africa: a mixed methods case study from Ghana. BMC Publ. Health.

[41] Masiga W.N., Munyua S.J.M. (2005). Global perspectives on animal welfare : Africa. Int Off Epizoot.

[42] Devereux S. (2014). http://www.ids.ac.uk/publications.

[43] Gibson T.J., Jackson E.L. (2017). The economics of animal welfare. Rev Sci Tech Off Int Epiz.

[44] Panel E., Ahaw W. (2011). Scientific Opinion concerning the welfare of animals during transport. EFSA J..

[45] Bulitta FS. Effects of Handling on Animals Welfare during Transport and Marketing. Department of energy and Technology.

[46] Schwartzkopf-Genswein K.S., Faucitano L., Dadgar S., Shand P., González L.A., Crowe T.G. (2012). Road transport of cattle, swine and poultry in North America and its impact on animal welfare, carcass and meat quality: a review. Meat Sci..

[47] Degeling C., Johnson J., Kerridge I. (2015). Implementing a One Health approach to emerging infectious disease: Reflections on the socio-political, ethical and legal dimensions. BMC Publ. Health.

[48] Pulido M.A., Estévez-Moreno L.X., Villarroel M., Mariezcurrena-Berasain M.A., Miranda-De la Lama G.C. (2019). Transporters knowledge toward preslaughter logistic chain and occupational risks in Mexico: an integrative view with implications on sheep welfare. J Vet Behav.

[49] Hoinville L.J., Alban L., Drewe J.A. (2013). Proposed terms and concepts for describing and evaluating animal-health surveillance systems. Prev. Vet. Med..

[50] Mitloehner F.M., Calvo M.S. (2008). Worker health and safety in Concentrated animal feeding operations. J. Agric. Saf. Health.

[51] Grandin T. (2019). The importance of stockmanship to maintain high standards of handling and transport of livestock and poultry. Livest Handl Transp.

